# *Phomopsis longanae* Chi-Induced Disease Development and Pericarp Browning of Harvested Longan Fruit in Association With Energy Metabolism

**DOI:** 10.3389/fmicb.2018.01454

**Published:** 2018-07-03

**Authors:** Yihui Chen, Hetong Lin, Shen Zhang, Junzheng Sun, Yifen Lin, Hui Wang, Mengshi Lin, John Shi

**Affiliations:** ^1^Institute of Postharvest Technology of Agricultural Products, College of Food Science, Fujian Agriculture and Forestry University, Fuzhou, China; ^2^Food Science Program, Division of Food System and Bioengineering, University of Missouri, Columbia, MO, United States; ^3^Guelph Food Research Center, Agriculture and Agri-Food Canada, Guelph, ON, Canada

**Keywords:** longan (*Dimocarpus longan* Lour.) fruit, *Phomopsis longanae* Chi, disease development, pericarp browning, energy metabolism, ATP content, energy charge, ATPase

## Abstract

Longan fruit is a popular subtropical fruit with a relatively short shelf life at room temperature mainly due to pericarp browning and fungal infection. This study aimed to investigate the infection of *Phomopsis longanae* Chi in longan fruit and its effects on the storability and shelf life of longan fruit. The relationship between the energy metabolism of harvested longan fruit and disease development and pericarp browning was elucidated. Results show that *P. longanae*-inoculation accelerated the deterioration of longan fruit and caused pericarp browning. It also led to the energy deficit in pericarp of longan fruit, which was reflected as lower contents of ATP and ADP, higher AMP content, and lower energy charge as compared to the control samples. Additionally, *P. longanae*-infection reduced the activities of H^+^-ATPase, Ca^2+^-ATPase, and Mg^2+^-ATPase in plasma, vacuolar, and mitochondrial membranes during the storage period. The results demonstrate that *P. longanae*-infection led to disease development and pericarp browning in harvested longan fruit, which were due to the infection-induced energy deficit and low ATPase activity that caused disorders of ion transport and distribution, and damaged the structure and function of vacuole, mitochondria, and eventually the whole cells of fruit tissues.

## Introduction

Longan is a popular subtropical fruit with a short shelf life at room temperature mainly due to pericarp browning and fungal infection (Holcroft et al., 2005; Chen et al., 2014; Zhang et al., 2017, 2018). Growing evidence suggests that the fruit tissue browning and loss of disease resistance are related to physiological disorders commonly caused by various stresses that can induce functional and structural damages of cellular membrane system (Yi et al., 2010; Jin et al., 2014; Li et al., 2017; Pan et al., 2017). Pathogenic infection, among various stress conditions, is a critical factor that can damage cell membrane in different ways such as creating energy deficit, oxidative burst, and alterations of membrane lipid compositions (Chen et al., 2014; Lin et al., 2017a; Sun et al., 2018; Zhang et al., 2018).

Membranes of plasma and organelles like vacuole and mitochondria are key components that contribute to cell integrity and prevent fruit tissue browning and disease development (Luo et al., 2012; Wang et al., 2016; Li D. et al., 2016; Lin Y.F. et al., 2016; Lin et al., 2017b, c, 2018). Plasma membrane is crucial for both cellular homeostasis and communications in extracellular environment (Olsen et al., 2009; Li D. et al., 2016). Whereas, vacuole and its membrane take part in regulating osmotic pressure, maintaining the homeostasis, and keeping internal phenolics from oxidase in cytoplasm which otherwise can lead to enzymatic browning (Holcroft et al., 2005; Anil et al., 2008; Lin et al., 2013, 2014, 2015, 2017b). Additionally, mitochondria play a foremost role in ATP production and thereby supply energy for normal life activities (Olsen et al., 2009; Lin et al., 2018), and the enzymes responsible for electron transfer and ATP synthesis are located on the inner membrane of mitochondria (Lin et al., 2017b, 2018). However, the regular function of the cell and these organelles depends on transmembrane transport of ions, in which active transport serves as an essential pathway with proton electrochemical potential gradient as a driving force (Morsomme and Boutry, 2000; Kasamo, 2003). Moreover, this driving force of transporting certain ions relies on energy from corresponding adenosine triphosphatase (ATPase) catalyzing ATP hydrolysis (Falhof et al., 2016). Previous literature indicated that hydrogen peroxide treatment could promote longan pericarp browning via decreasing the levels of ATP content and energy charge, and reducing activities of H^+^-ATPase, Ca^2+^-ATPase and Mg^2+^-ATPase in mitochondria, and damaging mitochondrial structure (Lin Y.X. et al., 2016; Lin et al., 2017b). In contrast, propyl gallate-retarded browning development in pericarp of harvested longans was resulted from retaining higher levels of ATP content and energy charge, as well as higher activities of mitochondrial ATPase (Lin et al., 2018). Furthermore, it was found that the acibenzolar-S-methyl treatment promoted the activities of Ca^2+^-ATPase and H^+^-ATPase in pear fruit, which enhanced its disease resistance against blue mold induced by *Penicillium expansum*-inoculation (Ge et al., 2017). Thus, the pathogenic infection-induced tissue browning and the reduction of disease resistance on longan fruit might be related to the damage of biomembranes via influencing energy status and ATPase activity.

*Phomopsis longanae* Chi is a major pathogenic fungus of harvested longan fruit in Southern China (Chen et al., 2014). Previous studies have shown that inoculation with pathogenic fungi on harvested longan fruit could lead to severe pericarp browning and disease development, which might be in association with elevated cell membrane permeability and lowered energy level (Chen et al., 2014; Zhang et al., 2017). However, more information is needed regarding changes in energy status and their damage to cellular membrane via affecting ATPase activity in pathogen-infected longan fruit. Therefore, the main goals of this work were to study the effects of the *P. longanae* infection on ATP, ADP, AMP, energy charge, and activities of H^+^-ATPase, Ca^2+^-ATPase, and Mg^2+^-ATPase in plasma, vacuolar, and mitochondrial membranes in pericarp of longan fruit, and investigate the effects of *P. longanae* infection on the disease development, pericarp browning, and the biomembrane damage of harvested longan fruit from a perspective of the changes in energy level and ATPase.

## Materials and Methods

### Materials and Treatments

*Phomopsis longanae* culturing and the preparation of spore suspension were conducted according to Chen et al. (2014). The concentration of spore suspension was diluted to 1 × 10^4^ spores mL^-1^ and used for inoculation.

“Fuyan” longan (*Dimocarpus longan* Lour. cv. Fuyan) fruit at commercial maturity were handpicked from a longan orchard (Quanzhou, Fujian, China). The harvested fruit were carefully packed and transported to a research laboratory in Fujian Agriculture and Forestry University within 3 h and stored at 4°C. Fruit in uniform maturity and size were selected for the experiment and any rotten or damaged fruit were excluded.

The fruit were washed with a sodium hypochlorite solution (0.5%) for 10 s to eliminate surface microorganisms, followed by being washed with sterile distilled water. The fruit samples were then air-dried. A total of 150 fruits were used for the analysis on harvest day (day 0). Another 3,000 longans were randomly divided into two groups (1,500 fruits each) for the following treatments: one group of 1,500 fruits was immersed in sterile deionised water for 5 min and defined as the control group, and the other group of 1,500 fruits was immersed in the *P. longanae* spore solution of 1 × 10^4^ spores mL^-1^ for 5 min. All fruits were then air dried and packed in a polyethylene bag with a thickness of 0.015 mm. Each bag contained 50 longan fruits and 30 bags were used for each treatment. The samples were then stored at 28°C with a relative humidity of 90%. For each treatment, three bags of fruit (total 150 longan fruits) were randomly selected on a daily basis during the storage period and used for the assessments of longan fruit. All the evaluations were conducted in triplicate.

### Assessments of the Index of Fruit Disease and Pericarp Browning

Longan fruit disease and pericarp browning were assessed based on our previous study (Chen et al., 2014). The lesion proportion on fruit surface of 50 individual longan fruits was measured and defined to five disease scales. The total browning area on inner pericarp of 50 selected longan fruits was measured and defined to six scales. The calculations of pericarp browning index and fruit disease index were performed based on the method of Chen et al. (2014).

### Measurement of ATP, ADP, and AMP and Energy Charge

The content of ATP, ADP, and AMP, and the energy charge were determined with 5 g of pericarp tissue from 10 longan fruits based on a previous study (Chen et al., 2014), using a high-performance liquid chromatography (HPLC, LC-2030C, Shimadzu Corporation, Kyoto, Japan) equipped with an ultraviolet detector and a Megres^TM^ C18 column (4.6 × 250 mm). Energy charge was calculated by (ATP+1/2 ADP)/(ATP+ADP+AMP).

### Assay of ATPase Activity

The activities of ATPase were measured following the methods of Lin et al. (2017b). Three enzymes (H^+^-ATPase, Ca^2+^-ATPase, and Mg^2+^-ATPase) from plasma membrane, vacuolar membrane and mitochondrial membrane were extracted respectively, from 1 g of pericarp tissue from 10 longan fruits. One unit of ATPase activity was considered as 1 μmol phosphorus released per minute at 660 nm. Bradford (1976) method was used to determine the protein content. The ATPase activity was expressed as U mg^-1^ protein.

### Statistical Analyses

All experiments were repeated three time and data were acquired. The values in figures were expressed in the format of the mean values and standard errors. Analysis of variance (ANOVA) was used to analyze the data using the software (SPSS version 17.0). Student’s *t*-test was used to compare the mean values of the data set. A *P*-value of less than or equal to 0.05 or 0.01 was considered statistically significant.

## Results

### Effects of *P. longanae* Infection on Indices of Fruit Disease and Pericarp Browning of Harvested Longan Fruit

**FIGURE 1:**
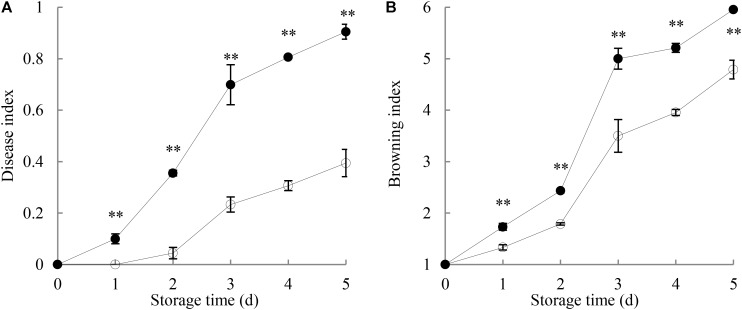
Effects of *P. longanae* infection on fruit disease index **(A)** and pericarp browning index **(B)** of harvested longan fruit during storage at 28°C. The asterisks indicate significant difference between control and *P. longanae*-inoculated fruit (^∗∗^*P* < 0.01). 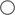, control; •, *P. longanae*-inoculation treatment.

**Figure 1A** shows that the disease index of harvested longan fruit increased with extending storage time. The disease lesions on *P. longanae*-inoculated longans developed quickly with white mycelia growing on the exocarp. By day 5 of the storage, the fruit disease index was 0.91, and the whole longan pericarp was covered with white lesions made of hypha. However, fruit disease index in control longans went up slowly (day 5 = 0.4). Further comparison shows that fruit disease index of *P. longanae*-inoculated longans were significantly (*P* < 0.01) higher as compared to the control samples during the storage period.

**Figure 1B** indicates that the pericarp browning index increased gradually in the first 2 days of storage, and then increased rapidly in the following days for both control samples and inoculated longans. The results of statistical analysis demonstrate that the browning index of *P. longanae*-inoculated longans were significantly (*P* < 0.01) higher than that of the control samples for the same storage time.

### Effects of *P. longanae* Infection on the Content of ATP, ADP, AMP, and Energy Charge in Pericarp of Harvested Longan Fruit

**FIGURE 2:**
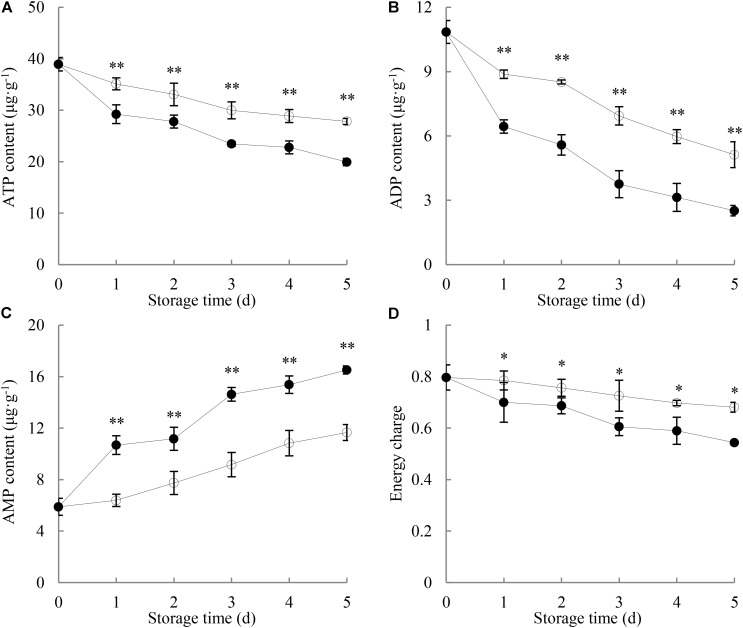
Effects of *P. longanae* infection on ATP **(A)**, ADP **(B)**, and AMP **(C)** contents and energy charge **(D)** in pericarp of harvested longan fruit during storage at 28°C. The asterisks indicate significant difference between control and *P. longanae*-inoculated fruit (^∗^*P* < 0.05, ^∗∗^*P* < 0.01). 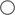, control; •, *P. longanae*-inoculation treatment.

As shown in **Figure 2A**, the ATP content in longans pericarp went down with increasing storage time. After 1 day of storage, the pericarp ATP content of *P. longanae*-inoculated longans displayed a drastic decrease from 29.2 μg g^-1^ (day 1) to 19.9 μg g^-1^ (day 5), while that of the control samples decreased slowly during the same storage period. On day 5 of the storage, the pericarp ATP content of control longans was 1.4 times higher than that of *P. longanae*-inoculated longans. Statistical analysis suggests that there was significant (*P* < 0.01) lower ATP content in the pericarp of *P. longanae*-inoculated longans than that of control fruit during storage day 1 to day 5.

**Figure 2B** illustrates that the ADP content in longans pericarp declined rapidly with increasing storage time. *P. longanae*-inoculated longans displayed lower content of pericarp ADP than the control longans during the whole storage period. After 5 days of storage, the ADP content in the pericarp of *P. longanae*-inoculated longans decreased from 10.85 to 2.52 μg g^-1^, while that of the control longans was at a value of 5.12 μg g^-1^. Statistical analysis indicates that there was significant (*P* < 0.01) lower pericarp ADP content in pericarp of *P. longanae*-inoculated longans from day 1 to day 5 of storage as compared to the control samples.

As shown in **Figure 2C**, the AMP content in pericarp of the control longans increased gradually in the whole storage period. Whereas, for the *P. longanae*-inoculated longans, it displayed a rapid rise during storage day 0 to day 1, changed slightly on storage day 2, followed by an increase from day 2 to day 5 of the storage. Further comparison reveals that the AMP content in pericarp of *P. longanae*-inoculated longans was significantly (*P* < 0.01) higher than that of control samples from the storage day 1 to day 5.

As displayed in **Figure 2D**, the energy charge in control longans decreased slowly as the storage time progressed, while that of the *P. longanae*-inoculated longans decreased much more quickly than the control longans. Further comparison shows that *P. longanae*-inoculated longan pericarp had significant (*P* < 0.05) lower energy charge than the control samples during storage period.

### Effects of *P. longanae* Infection on H^+^-ATPase Activities in Membranes of Plasma, Vacuole and Mitochondria in Pericarp of Harvested Longan Fruit

**FIGURE 3:**
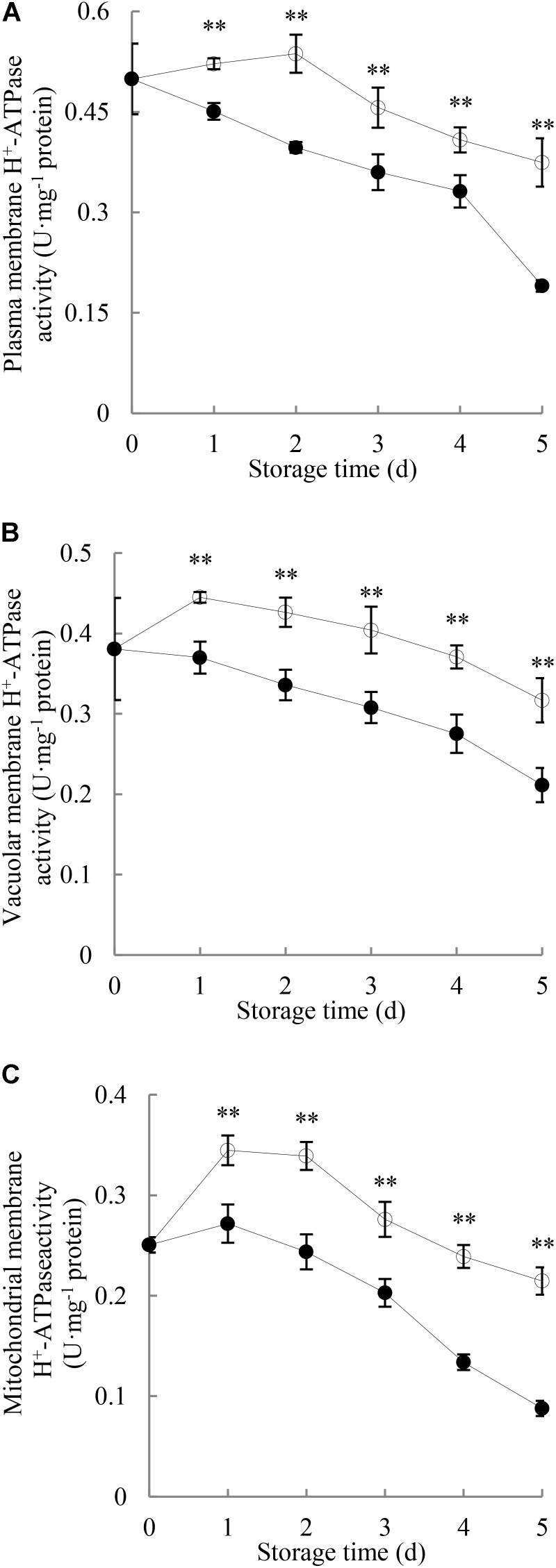
Effects of *P. longanae* infection on activities of H^+^-ATPase in membranes of plasma **(A)**, vacuole **(B)**, and mitochondria **(C)** in pericarp of harvested longan fruit during storage at 28°C. The asterisks indicate significant difference between control and *P. longanae*-inoculated fruit (^∗∗^*P* < 0.01). 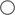, control; •, *P. longanae*-inoculation treatment.

**Figure 3A** illustrates that the H^+^-ATPase activity in plasma membrane of the control longans pericarp grew slightly in the first 2 days of the storage and then decreased; while in the *P. longanae*-inoculated longans fruit, it displayed a quick decrease during the first 4 days, followed with a sharp decline from the fourth to the fifth day. Further comparison shows that the plasma membrane H^+^-ATPase activity in pericarp of *P. longanae*-inoculated longans was significantly (*P* < 0.01) lower than that of the control samples during the whole storage period.

As shown in **Figure 3B**, H^+^-ATPase activity in vacuolar membrane of control longan pericarp increased quickly on the first day of storage, and then diminished gradually from the first day to day 5 of the storage. However, H^+^-ATPase activity in vacuolar membrane of *P. longanae*-inoculated longan pericarp exhibited a sharp decrease during storage. Further comparison demonstrates that the vacuolar membrane H^+^-ATPase activity in pericarp of *P. longanae*-inoculated longans was significantly (*P* < 0.01) lower as compared with the control group in the whole storage period.

As shown in **Figure 3C**, H^+^-ATPase activity in mitochondrial membrane of control longan pericarp went up rapidly on the first day, then decreased slightly on the second day, and continued to drop rapidly to the last storage day. Whereas, the H^+^-ATPase activity in mitochondrial membrane of *P. longanae*-inoculated longan pericarp exhibited a mild increase on the first day of storage, and then diminished rapidly in the following days. Further statistical comparison indicates that H^+^-ATPase activity in mitochondrial membrane of pericarp of *P. longanae*-inoculated longans was significantly (*P* < 0.01) lower than that of the control samples from the first to the last day of storage.

### Effects of *P. longanae* Infection on Ca^2+^-Atpase Activities in Membranes of Plasma, Vacuole and Mitochondria in Pericarp of Harvested Longan Fruit

**FIGURE 4:**
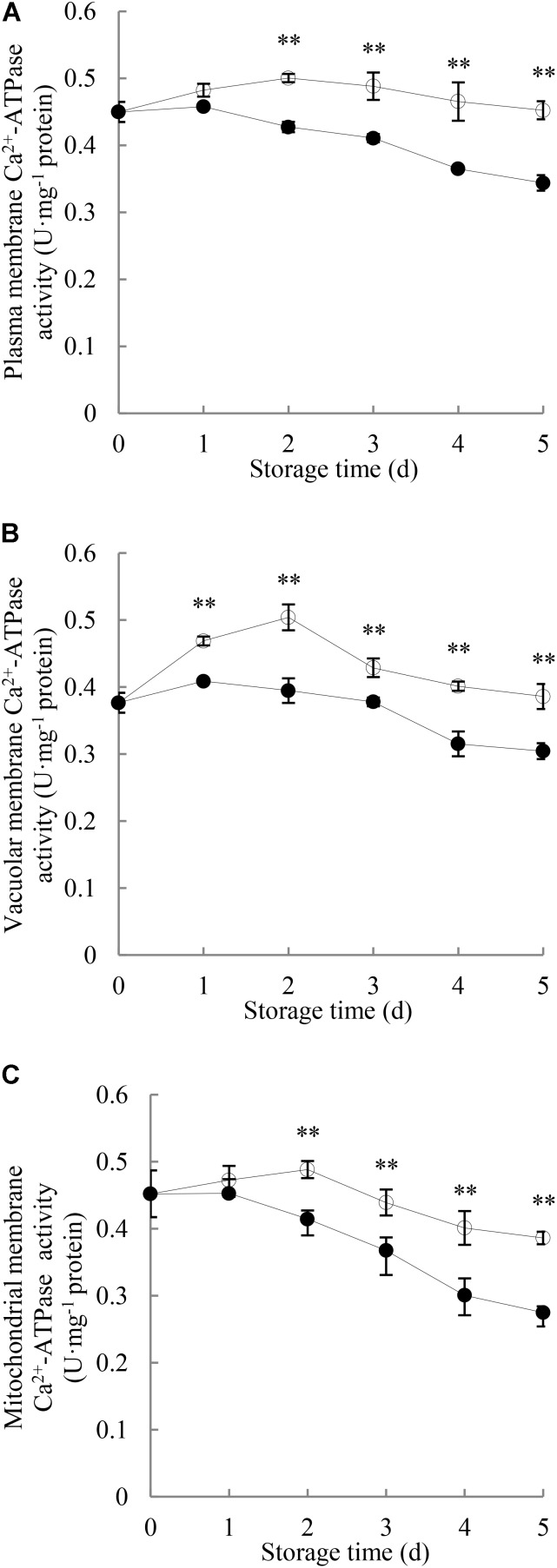
Effects of *P. longanae* infection on activities of Ca^2+^-ATPase in membranes of plasma **(A)**, vacuole **(B)**, and mitochondria **(C)** in pericarp of harvested longan fruit during storage at 28°C. The asterisks indicate significant difference between control and *P. longanae*-inoculated fruit (^∗∗^*P* < 0.01). 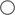, control; •, *P. longanae*-inoculation treatment.

As shown in **Figure 4A**, Ca^2+^-ATPase activity in plasma membrane of the control longan pericarp increased to a small degree during the first 2 days and then decreased gradually, while it declined as storage time progressed in the *P. longanae*-inoculated longan pericarp. Statistical analysis indicates that there were significant (*P* < 0.01) differences in the plasma membrane Ca^2+^-ATPase activities between the pericarp of *P. longanae*-inoculated and control fruit from day 2 to day 5.

As shown in **Figure 4B**, Ca^2+^-ATPase activity in vacuolar membrane of control longan pericarp increased from 0.37 U mg^-1^ protein on day 0 of storage to a maximum value of 0.51 U mg^-1^ protein on storage day 2, but then declined gradually to 0.38 U mg^-1^ protein on storage day 5. Whereas, the *P. longanae*-inoculated longans showed a slow increase in the Ca^2+^-ATPase activity in vacuolar membrane in the first day of the storage period and then decreased. Statistical comparison suggests that there was significantly (*P* < 0.01) lower Ca^2+^-ATPase activity in vacuolar membrane of pericarp of the *P. longanae*-inoculated longans than that of the control samples during day 1 to day 5 of the storage.

The Ca^2+^-ATPase activity in mitochondrial membrane in longans pericarp (**Figure 4C**) followed a similar trend as Ca^2+^-ATPase activity in plasma membrane (**Figure 4A**), and the mitochondria membrane Ca^2+^-ATPase activity in pericarp of *P. longanae*-inoculated longans were notably (*P* < 0.01) lower than that of the control longans during the last 3 days of storage.

### Effects of *P. longanae* Infection on Mg^2+^-Atpase Activities in the Membrane of Plasma, Vacuole and Mitochondria in Pericarp of Harvested Longan Fruit

**FIGURE 5:**
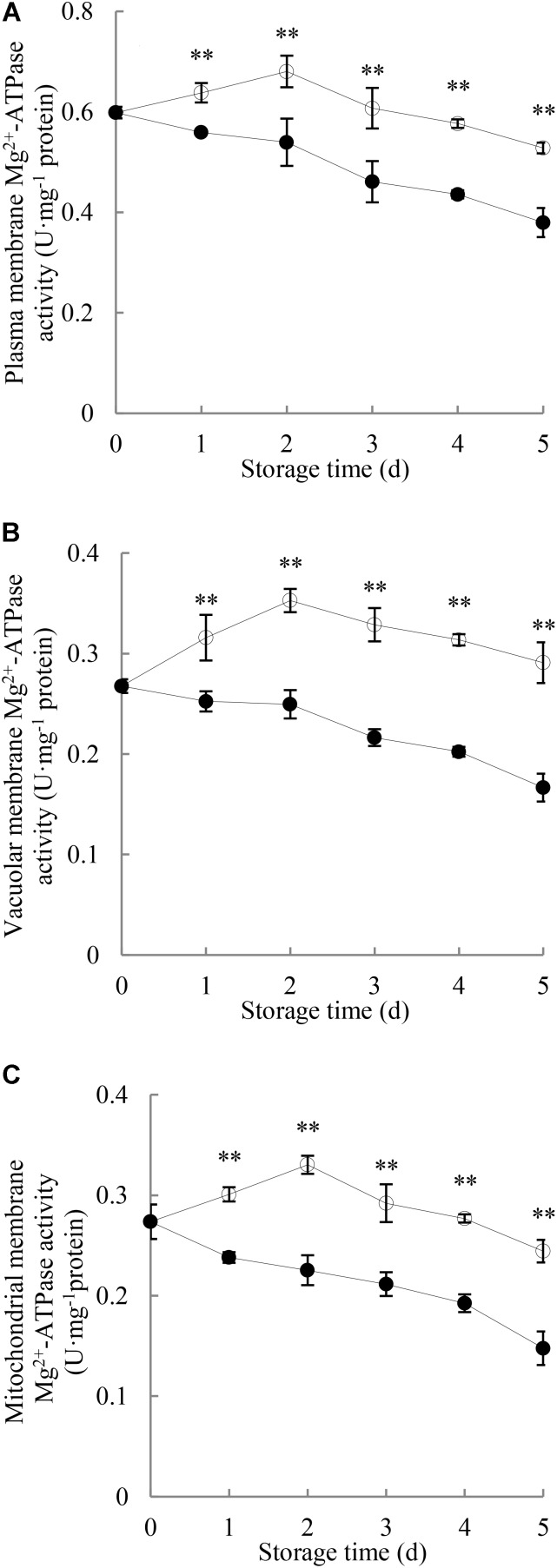
Effects of *P. longanae* infection on activities of Mg^2+^-ATPase in membranes of plasma **(A)**, vacuole **(B)**, and mitochondria **(C)** in pericarp of harvested longan fruit during storage at 28°C. The asterisks indicate significant difference between control and *P. longanae*-inoculated fruit (^∗∗^*P* < 0.01). 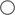, control; •, *P. longanae*-inoculation treatment.

As shown in **Figure 5**, changes in Mg^2+^-ATPase activity in the membranes of plasma, vacuole and mitochondria of pericarp during the entire period of storage were observed. The Mg^2+^-ATPase activity in membranes of plasma, vacuole and mitochondria of the pericarp of the control longans rose rapidly toward a maximum on day 2 and then declined. *P. longanae*-inoculated longans showed significant (*P* < 0.01) lower pericarp Mg^2+^-ATPase activity during the whole storage period than that of the control longans.

## Discussion

### The Role of Energy Deficit in *P. longanae*-Induced Pericarp Browning and Disease Development of Harvested Longan Fruit

ATP generated from mitochondria is the most important energy source for life activities (Pan et al., 2017). In plant cells, ATP is used for the synthesis of fatty acids, phospholipids, and proteins on membranes of the cell and organelles such as vacuole and mitochondria to help sustain their structure and regular function (Jin et al., 2014; Zhou et al., 2014). However, ATP synthesis and energy level of fruit can be reduced by postharvest stress conditions like pathogen infection via altering respiration pathway and inducing excessive accumulation of reactive oxygen species (ROS), which can weaken mitochondria and result in energy deficit (Yi et al., 2008; Chen et al., 2014; Zhang et al., 2017). Recent studies indicated that the energy deficit was a critical factor leading to the damage of cellular membranes including decompartmentalization that conduced to enzymatic browning (Duan et al., 2004; Jiang et al., 2004; Jin et al., 2014; Lin Y.X. et al., 2016; Lin et al., 2017b). Other than membrane damage, insufficient energy supply may give rise to the reduction of disease resistance by inactivating defensive responses like the synthesis of pathogenesis-related protein (Qu et al., 2008; Yi et al., 2009; Chen et al., 2014). Zhang et al. (2017, 2018) reported that *Lasiodiplodia theobromae-*infection could reduce the energy charge and damage the membrane structure in pericarp of inoculated longan fruit while aggravate disease progress and pericarp browning. Besides, exogenous ATP supply was beneficial for keeping membrane integrity to decrease pericarp browning and disease development of litchi and longan fruit (Song et al., 2006; Yi et al., 2008, 2009; Chen et al., 2015; Lin et al., 2017a).

The data acquired from this work indicate that *P. longanae*-inoculated longans showed drastic increase in the indices of pericarp browning and fruit disease with notably higher values as compared to the control samples during 5 days of storage (**Figure 1**). In the meanwhile, the energy charge and content of ATP and ADP in the pericarp of *P. longanae*-inoculated samples decreased quickly and were much lower than those of the control fruit (**Figures 2A,B,D**). The pericarp AMP content of inoculated longans exhibited an uptrend at relative higher levels with contrast to the control fruit throughout the storage period (**Figure 2C**). Correlation analysis suggests that there was an obvious inverse correlation between fruit disease index and both pericarp ATP content (*r* = -0.919, *P* < 0.01) and energy charge (*r* = -0.963, *P* < 0.01) in longans inoculated with *P. longanae*. The correlation analysis also denotes that the pericarp browning index were in an inverse correlation with ATP content (*r* = -0.917, *P* < 0.05) and energy charge (*r* = -0.968, *P* < 0.01) in pericarp of *P. longanae*-inoculated longans, respectively. However, the pericarp AMP content of inoculated longans was positively correlated with either fruit disease index (*r* = 0.952, *P* < 0.01) or pericarp browning index (*r* = 0.955, *P* < 0.01). These results provide convincing evidence that accelerated disease development and pericarp browning of inoculated longan fruit is closely associated with the infection-induced energy deficit. These findings were in agreement with our previous study (Chen et al., 2014).

### The Role of ATPase in *P. longanae*-Induced Pericarp Browning and Disease Development of Harvested Longan Fruit

ATPase like H^+^-ATPase, Ca^2+^-ATPase, and Mg^2+^-ATPase take vital roles in botanic cellular homeostasis and physiological metabolisms as they are located in the membranes of cell and organelles such as mitochondria and vacuole, and catalyze ATP hydrolysis for transmembrane transport of corresponding ions and signal transmission (Elmore and Coaker, 2011; Wang et al., 2015; Liu et al., 2016; Falhof et al., 2016; Pan et al., 2017). The distribution of H^+^ not only affects cellular pH value, but also is the key part of transmembrane electrochemical gradient and electrodynamic potential, which have great influence on various kinds of physiological activities, especially on the respiratory chain and ATP synthesis (Morsomme and Boutry, 2000; Olsen et al., 2009; Falhof et al., 2016; Li P.Y. et al., 2016; Li et al., 2017). Ca^2+^ acts as the second-messenger in signals transmission, while transport and distribution disorders of Ca^2+^ might cause structural damage of cellular membranes and metabolic dysregulation (Muchhal et al., 1997; Li et al., 2017; Pan et al., 2017). In addition, Mg^2+^ acts as a cofactor in respiratory and energy metabolisms in fruit cells, and its disorder will conduce to peroxidation and oxidative stress (Tewari et al., 2006; Nozadze et al., 2015). Besides, balanced Ca^2+^ and Mg^2+^ distribution and transport are beneficial for keeping cellular osmotic pressure, which contributes to cell structural integrity (Wang et al., 2008; Li et al., 2017). Thus, the regular activity of ATPase can maintain the dynamic equilibrium of these ions and transmembrane electrochemical gradient to support the homeostasis and integrity of botanic cell, as well as physiological activities relating to disease-resistant responses (Wang et al., 2015; Lin et al., 2017b, 2018). However, the dysfunction of ATPase may break the endo-cellular homeostasis and damage plasma, vacuolar, and mitochondrial membranes, and thereby cause energy deficit and integrity loss, result in tissue browning and weakened disease resistance (Morsomme and Boutry, 2000; Anil et al., 2008; Jin et al., 2014; Shi et al., 2015; Li et al., 2017; Pan et al., 2017). Furthermore, the change in ATPase activity of harvested fruit might be associated with energy status and pathogenic stress. Wang et al. (2008) reported that the depletion of ATP was associated with pathogen infection in the *Physalospora piricola* Nose-inoculated “Ya” pear fruit, and the activity of Ca^2+^-ATPase in pulp of inoculated pears declined and the decline rate was faster than the control (Wang et al., 2008). Treatment with nitric oxide maintained higher activities of Ca^2+^-ATPase in harvested peach fruit during storage, which was in association with the inhibition on disease development of *Monilinia fructicola*-inoculated fruit (Shi et al., 2015).

This study shows that compared with the control longan fruit, the H^+^-ATPase, Ca^2+^-ATPase, Mg^2+^-ATPase activities in *P. longanae*-inoculated longan fruit were lower and reduced continuingly during the storage (**Figures 3–5**), as the indices of fruit disease and pericarp browning kept increasing with higher values (**Figure 1**) and the pericarp ATP content and energy charge declined gradually to lower levels (**Figures 2A,D**). The results indicate that the accelerated pericarp browning and loss of disease resistance of longan fruit during storage could be attributed to energy deficit and inactivity of ATPase. The low H^+^-ATPase activity caused by infection and energy deficit could induce cellular pH value turbulence to threaten homeostasis. Besides, it could also lower ATP synthesis in turn via reducing the proton electrochemical gradient, which was in accordance with the decrease in ATP content in pericarp of *P. longanae*-inoculated fruit as illustrated in **Figure 2A**. Furthermore, the energy deficit and decreased activities of Ca^2+^-ATPase and Mg^2+^-ATPase led to incapability of pumping out redundant Ca^2+^ and Mg^2+^ from cytoplasm or transferring them to vacuole and mitochondria. This contributed to the disequilibrium of Ca^2+^ and Mg^2+^ distribution either in cytoplasm or between endo-cellular and extracellular environment, resulting in disturbed calcium messenger system, respiratory metabolism, and transmembrane osmotic pressure. These disorders aggravated the energy deficit and the homeostasis by damaging the structure of vacuole, mitochondria, and the whole cell, which jointly conduced to the pericarp browning and loss of disease resistance of *P. longanae*-inoculated longan fruit.

## Conclusion

In summary, this work demonstrate that *P. longanae*-inoculation could reduce the ATP and ADP content, but increase AMP content, and thereby lower the energy charge, cause energy deficit in pericarp of *P. longanae*-infected longans. Consequently, the activities of H^+^-ATPase, Ca^2+^-ATPase, and Mg^2+^-ATPase in the membrane of plasma, vacuole, and mitochondria decreased, which aggravated the structural and functional damage of cellular biomembrane system and energy deficit, leading to disease development and pericarp browning of *P. longanae* -infected longan fruit.

## Author Contributions

YC and HL designed the research. SZ, JS, YL, and HW conducted the experiments and analyzed the data. YC and SZ wrote the manuscript. HL revised the manuscript. ML and JS edited English language of the manuscript. All authors have approved the submission and publication of the manuscript.

## Conflict of Interest Statement

The authors declare that the research was conducted in the absence of any commercial or financial relationships that could be construed as a potential conflict of interest.
